# The characteristics of acute macular neuroretinopathy following COVID-19 infection

**DOI:** 10.1186/s12886-024-03283-2

**Published:** 2024-01-10

**Authors:** Hui Feng, Meng Zhao, Jing Mo, Xusheng Cao, Weixin Chen, Hong Wang

**Affiliations:** Beijing Ophthalmology and Visual Sciences Key Laboratory, Beijing Tongren Hospital, Beijing Tongren Eye Center, Beijing Institute of Ophthalmology, Capital Medical University, 1 Dongjiaominxiang Street, Dongcheng District, Beijing, China

**Keywords:** Severe acute respiratory syndrome coronavirus-2, COVID-19, Acute macular neuroretinopathy, Optical coherence tomography, Optical coherence tomography angiography, Visual field

## Abstract

**Background:**

In this study, we report a case series of acute macular neuroretinopathy (AMN) associated with COVID-19 infection.

**Methods:**

This retrospective observational study was conducted at Beijing Tongren Hospital. We reviewed patients who were diagnosed with AMN within one month of testing positive for COVID-19 using real-time reverse transcription-polymerase chain reaction (RT-PCR).

**Results:**

A total of 11 AMN patients (20 eyes) were included in the study. The mean age was 33.8 ± 12.6 years. The average interval between a positive COVID-19 PCR test and the onset of ocular symptoms was 2.8 ± 2.5 days. The mean follow-up period for the patients was 12.5 ± 3.8 weeks. Imaging characteristics of AMN patients following COVID-19 infection included areas of low reflectivity on near-infrared reflectance (NIR) imaging, hyperreflective lesions at the level of the outer plexiform layer (OPL) and outer nuclear layer (ONL) and disruption of the ellipsoid zone (EZ) on spectral domain optical coherence tomography (SD-OCT) B-scans. Visual field examinations revealed parafoveal scotomas that closely corresponded to the clinical lesions. Optical coherence tomography angiography (OCT-A) demonstrated impaired perfusion in the deep retinal vascular plexus. Fluorescein angiography (FA), indocyanine green angiography (ICGA), and spontaneous fundus autofluorescence showed no significant abnormalities. During follow-up, partial improvement in retinal lesions was observed in NIR imaging and SD-OCT in some patients, but a proportion of patients still exhibited persistent retinal damage and no improvement in visual field scotomas.

**Conclusion:**

COVID-19-related AMN share similar clinical and imaging features with AMN due to other causes, as evidenced by the persistent presence of visual field scotomas over a longer duration.

**Trail registration:**

https://www.chictr.org.cn/; identifier: ChiCTR2100044365

## Background

As the result of the first reported cases of severe illness and pneumonia in Wuhan, China, the World Health Organization declared “coronavirus disease 2019 (COVID-19)” as the novel severe acute respiratory syndrome coronavirus (SARS-CoV-2) [1]. As well as its deadly respiratory syndrome, COVID-19 can also cause multiple organ complications due to inappropriate immune and inflammatory responses, endothelial dysfunction, and thromboembolic diseases [2]. An increased level of hypercoagulability can cause thrombus formation, resulting in localized microvascular occlusions. There are also a variety of pathological events contributing to pro-inflammatory and anti-fibrinolytic conditions. Several factors have been linked to hypercoagulability in COVID-19 patients, including an increased level of D-dimer and the development of antiphospholipid antibodies [3].

Acute macular neuroretinopathy (AMN) is a rare disease affecting the outer layers of the retina, diagnosed through multimodal imaging [4]. Fundus photographs typically reveal petaloid-shaped dark red lesions around the central macula. The characteristic features include: (1) areas of low reflectivity adjacent to the macula on near-infrared reflectance (NIR) imaging, (2) hyperreflective lesions at the level of the outer plexiform layer (OPL) and outer nuclear layer (ONL) and disruption of the ellipsoid zone (EZ) on optical coherence tomography (OCT) B-scans, consistent with the observed lesions on NIR imaging [5]. The disease usually follows a self-limited course, with gradual recovery, while the ONL tends to become thinner [6]. Despite being unclear about the cause, AMN is believed to be an acute lesion of the outer retina caused by vascular dysregulation in the deep retinal plexus, while some contend that there is a disruption in the flow of blood in the choroid [7]. There have actually been studies linking AMN to vascular factors (hypotension or hypertension, use of sympathomimetic medications, anaphylactic shock, thrombocytopenia, anemia, hyperlipidemia, hypovolemia, and dehydration) as well as optic neuritis, and infectious such as dengue fever, influenza viruses infections, and vaccinations [8].

In 2022, during the most severe stage of the SARS-CoV-2 pandemic in Beijing, a marked rise in AMN cases was observed at Beijing Tongren Hospital. In this study, we report on 11 patients who developed AMN following COVID-19 infection, exploring the characteristics, patterns of occurrence, and possible mechanisms of AMN after COVID-19 infection.

## Methods

This is a retrospective observational study conducted at Beijing Tongren Hospital. The study included patients diagnosed with AMN and a history of COVID-19 infection within one month from December 1, 2022, to February 28, 2023. The study was approved by the Institutional Research and Ethics Committee of Beijing Tongren Hospital and followed the principles of the Helsinki Declaration.

The inclusion criteria for this study were as follows: (1) Patients diagnosed with AMN exhibiting areas of low reflectivity adjacent to the macula on NIR imaging, hyperreflectivity in the OPL and disruption of the photoreceptor layer on spectral domain optical coherence tomography (SD-OCT) B-scans [5]; (2) Positive real-time reverse transcription polymerase chain reaction (RT-PCR) for SARS-CoV-2 obtained from nasopharyngeal swabs; (3) Close temporal correlation between the onset of the disease and the confirmed SARS-CoV-2 infection within one month.

Demographic data of the AMN cases were collected, including gender, age, the existence of systemic diseases (such as hypertension and diabetes), and the usage of oral drugs (especially contraceptives). Data of ophthalmic examination was also collected at baseline and during the subsequent follow-up period. All patients underwent comprehensive ocular examinations, including best-corrected visual acuity (BCVA) (Snellen visual acuity charts), anterior segment examination with a slit-lamp (BQ900, Haag-Streit, Bern, Switzerland), intraocular pressure (IOP) (NT510, Non-Contact tonometer, Nidek, Gamagori, Japan), and indirect ophthalmoscopy. In addition, relevant imaging studies were performed, including fundus photography (CR-DGi Non-mydriatic retinal camera, Canon, Tokyo, Japan), B-scan ultrasound (Echoscan, US-400; Nidek Co Ltd, Gamagori, Japan), NIR, fluorescein angiography (FFA), indocyanine green angiography (ICGA) (Spectralis HRA; Heidelberg Engineering, Inc, Heidelberg, Germany), SD-OCT (RTVue, Optovue, Inc, CA) and optical coherence tomography angiography (OCT-A) (RTVue AngioVue; Optovue Inc.). Blood tests were conducted for all patients to exclude infection, and relevant laboratory investigations were performed to evaluate systemic conditions.

All patients had complete medical records, including gender, age, medical history, the results of various imaging techniques and their conditions at the time of their last visit. All statistical data in this study were entered into Excel. The continuous variables were described as means and standard errors of the mean (SEM). The categorical variables were described as frequencies and constituent ratios.

## Result

This case series, based on a retrospective observational study conducted at the hospital, included 20 eyes from 11 patients. The majority of the patients were female (male-to-female ratio = 0.57:1). The average age at the first onset of symptoms was 33.8 ± 12.6 years. (Table 1) One pediatric AMN patient, a 16-year-old male, was identified in this study. The average time interval between positive COVID-19 PCR testing and the onset of ocular symptoms was 2.8 ± 2.5 days, with a minimum of 1 day and a maximum of 10 days. The average follow-up duration for the patients was 12.5 ± 3.8 weeks, ranging from 8 to 20 weeks. All the patients included in our study completed the last dose of the COVID-19 vaccine between June 2021 and April 2022. The diagnosis of AMN occurred at least 6 months after the last COVID-19 vaccine dose. Our patient had no history of oral contraceptives or other medications.

**Table 1:** Demographics and disease characteristics for patients with AMN

Finding	Number (% of total)
Age	33.8 ± 12.6
Sex	
Male	4 (36.3%)
Female	7 (63.6%)
Symptoms	
Scotomas	6 (54.5%)
Decreased vision	3 (27.3%)
Blurry vision	5 (45.5%)
Laterality	
Bilateral	9 (81.8%)
Unilateral	2 (18.2%)

A total of 81.8% of the patients had bilateral AMN. All AMN patients reported visual symptoms, including 6 cases (54.3%) with central or paracentral scotomas, 3 cases (27.3%) with visual acuity decline, and 5 cases (45.5%) with blurred vision. Three patients experienced a combination of two symptoms. (Table 1) At the initial visit, the BCVA of 16 eyes (80.0%) was 20/40 or better, while 3 eyes (15.0%) had a BCVA of 20/200 or worse. In the final follow-up, all patients achieved a BCVA of 20/40 or better, but 9 patients (81.8%) continued to experience persistent scotomas. Table 2 provides detailed demographic data of all patients with clinical features.

**Table 2:** Demographics, clinical findings and outcome for patients with AMN

Number	Age	Sex	Laterality and quality of symptoms	Time to onset of visual symptoms after COVID RT-PCR positive	BCVA at present	Clinical Findings						BCVA at last follow-up	Outcome	Follow-up days
						NIR	SD-OCT	OCTA	ICGA	FFA	FAF			
1	34	Male	Paracentral scotoma in LE.	2 days	R:20/20 L:20/40	Sharply demarcated dark lesions.	Disruption of the underlying ellipsoid zones.	Decreased vascular flow signal in the deep capillary plexus.	/	/	Normal.	R:20/20 L:20/20	Scotoma still present; ellipsoid zones were partially reversed.	8 weeks
2	42	Male	Blurred vision in both eyes.	3 days	R:CF L:20/100	Lesions were not obvious.	Disruption of the underlying ellipsoid zones and outer retinal hyper-reflectivity.	Decreased vascular flow signal in the deep capillary plexus.	Normal.	Normal.	Normal.	R:20/20 L:20/20	Visual acuity improved but scotoma still present; disruption of the underlying ellipsoid and interdigitation zones partially reversed.	12 weeks
3	37	Male	Scotomas in central visual field in LE.	10 days	R:20/20 L:20/25	Dark grey lesion.	Disruption of the underlying ellipsoid and interdigitation zones.	/	Scattered small patches of low fluorescence can be seen in the late stage.	Normal.	Normal.	R:20/20 L:20/20	Visual acuity improved but scotoma still present; ellipsoid and interdigitation zones were partially reversed.	12 weeks
4	39	Female	Blurred vision and paracentral scotoma in both eyes.	1 days	R:20/20 L:20/32	Dark grey lesion.	Disruption of the underlying ellipsoid and interdigitation zones.	Decreased vascular flow signal in the deep capillary plexus.	/	/	Normal.	R:20/20 L:20/20	Visual acuity improved but scotoma still present; disruption of the underlying ellipsoid and interdigitation zones not improved.	16 weeks
5	32	Female	Blurred vision and paracentral scotoma in both eyes.	2 days	R:20/25 L:20/25	Sharply demarcated dark lesions.	Disruption of the underlying ellipsoid and interdigitation zones.	Decreased vascular flow signal in the deep capillary plexus.	Normal.	Normal.	Normal.	R:20/25 L:20/20	Visual acuity improved but scotoma still present; disruption of the underlying ellipsoid and interdigitation zones partially reversed.	8 weeks
6	34	Female	Blurred vision in both eyes.	3 days	R:20/25 L:20/20	Lesions were not obvious.	Disruption of the underlying ellipsoid and interdigitation zones.	Decreased vascular flow signal in the deep capillary plexus.	/	/	Normal.	R:20/20 L:20/20	Visual acuity improved; disruption of the underlying ellipsoid and interdigitation zones not improved.	12 weeks
7	21	Female	Decreased vision in both eyes.	1 days	R:20/20 L:20/400	Round, dark lesions in the fovea.	Disruption of ellipsoid zone and hyper-reflectivity of the outer nuclear layer.	Decreased vascular flow signal in the deep capillary plexus.	Normal.	Normal.	Normal.	R:20/20 L:20/20	Visual acuity improved but scotoma still present; disruption of the underlying ellipsoid not improved.	15 weeks
8	16	Male	Decreased vision in both eyes.	2 days	R:20/25 L:20/800	Grayish lesions in the fovea.	Disruption of ellipsoid zone and hyper-reflectivity of the outer nuclear layer.	/	Normal.	Normal.	Normal.	R:20/20 L:20/25	Visual acuity improved but scotoma still present; disruption of the underlying ellipsoid and interdigitation zones partially reversed.	12 weeks
9	40	Female	Blurred vision and central scotoma in both eyes.	2 days	R:20/20 L:20/20	Dark grey, sharply defined oval lesions.	Disruption of the underlying ellipsoid and interdigitation zones.	Decreased vascular flow signal in the deep capillary plexus.	Normal.	Normal.	Normal.		scotoma still present; disruption of the underlying ellipsoid and interdigitation zones partially reversed.	20 weeks
10	36	Female	Central scotoma in both eyes.	2 days	R:20/20 L:20/20	Well- demarcated, petalloid-shaped, dark grey macular lesions.	Disruption of ellipsoid zone and hyper-reflectivity of the outer nuclear layer.	Decreased vascular flow signal in the deep capillary plexus.	/	/	Normal.		scotoma still present; disruption of the underlying ellipsoid and interdigitation zones partially reversed.	15 weeks
11	41	Female	Decreased vision in both eyes.	3 days	R:20/25 L:20/40	Dark grey lesion.	Disruption of ellipsoid zone and hyper-reflectivity of the outer nuclear layer.	/	Scattered small patches of low fluorescence can be seen in the early and late stage.	Normal.	Normal.		Visual acuity improved; disruption of the underlying ellipsoid and interdigitation zones partially reversed; outer nuclear layer atrophy.	8 weeks

Abbreviations: BCVA, best corrected visual acuity; IOP, intraocular pressure; RE, right eye; LE, left eye; HM, hand movement; CF, counting finger; SD-OCT, spectral domain optical coherence tomography; OCTA, optical coherence tomography angiography; NIR, near-infrared reflectance; FFA, fundus fluorescein angiography; ICGA, indocyanine green angiography; FAF, fundus autofluorescence

**Fig. 1:**
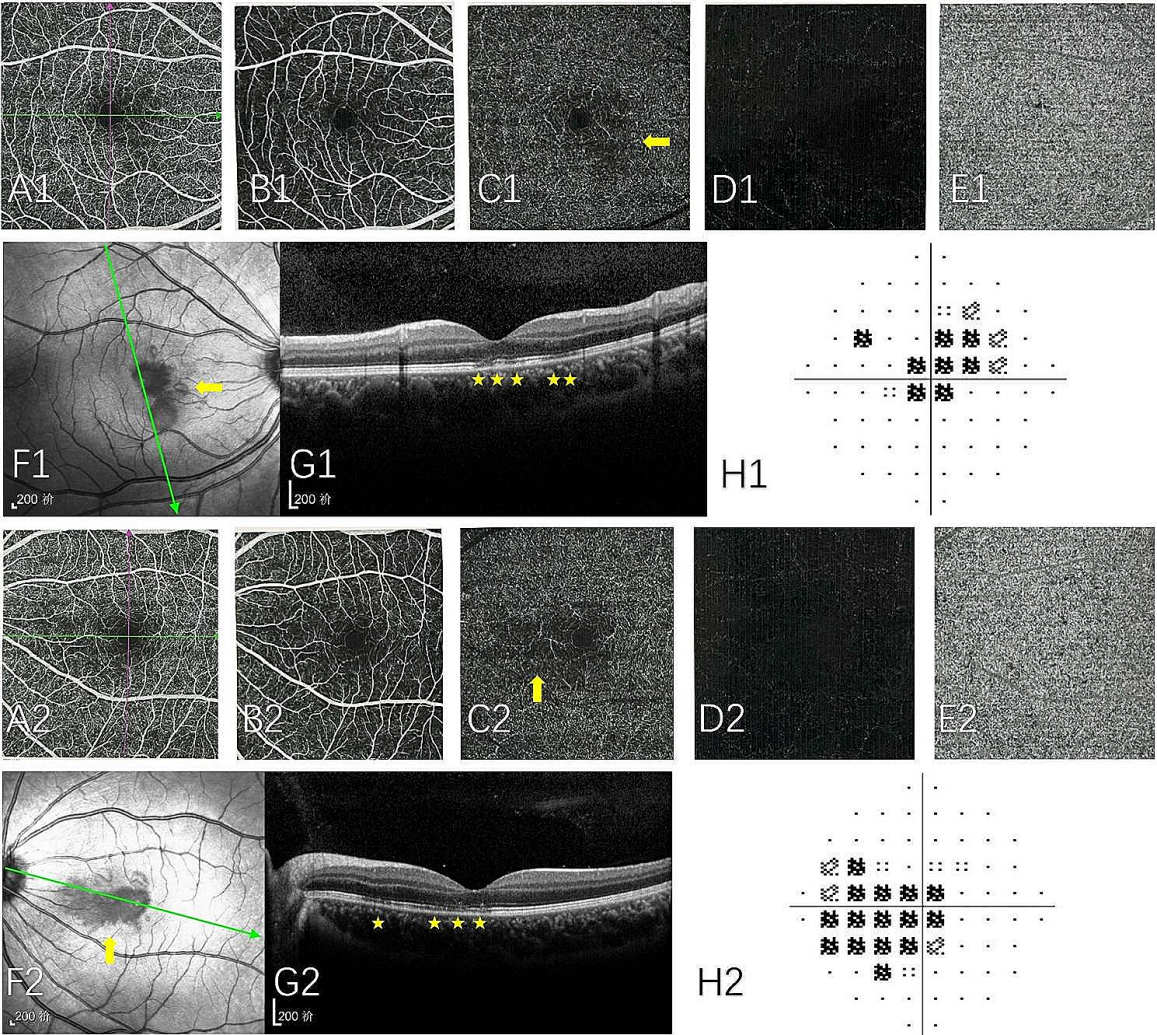
This figure demonstrates the ocular examination results of the patient 5. Number 1 represents the patient’s right eye result, and number 2 represents the left eye result. Optical coherence tomography angiography (OCTA) superficial capillary plexus and intermediate capillary plexus “en-face” showing normal flow (**A** and **B**). OCTA deep capillary plexus “en-face” showing areas of reduced flow in the areas of decreased reflectivity surrounding the fovea on near infrared images (**C**). OCTA avascular zone and choriocapillary plexus “en-face” showing normal flow (**D** and **E**). The petaloid lesions are well visualized as areas of decreased reflectivity surrounding the fovea (yellow arrow) on near infrared images (**F**). The SD-OCT scans show attenuation of ellipsoid zone reflectivity (yellow pentagram) of the both eyes (**G**). Humphrey 10/2 visual field test showed center scotomas (**H**)

**Fig. 2:**
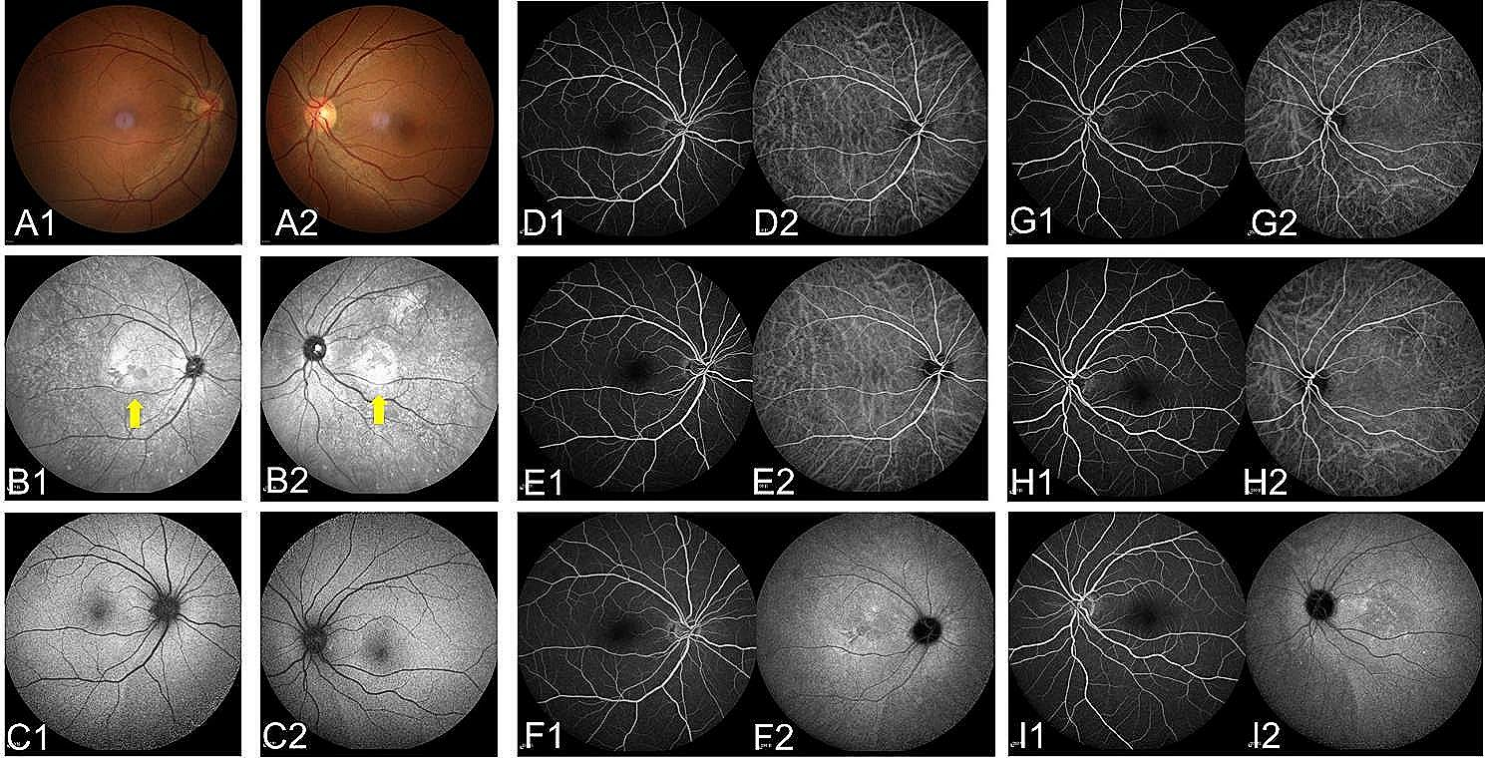
The figure demonstrates the ocular examination results of the patient 8. Number 1 represents the patient’s right eye result, and number 2 represents the left eye result. Obvious abnormalities were not found in fundus photographs (**A**1 and 2) and fundus autofluorescence (FAF) examination (**C**1 and 2) in both eyes. Infrared reflectance (IR) imaging revealed distinct grayish lesions (**B**1 and 2) (yellow arrow). No abnormal fluorescence was observed in the early-phase (**D** and **G**), middle-phase (**E** and **H**) and late-phase (**F** and **I**) of FA and ICGA images of the AMN patients, which appeared to be normal

NIR imaging was performed on all 11 patients. We observed distinct NIR abnormalities in 9 patients. The AMN lesions in each patient were described as being grayish with distinct edges. (Fig. 1F1, F2 and Fig. 2B1, B2). During the follow-up, corresponding clinical changes in the NIR abnormalities were noted. Among the 11 eyes of 6 patients, the NIR lesions showed slight reduction in size, while in the 6 eyes of 3 patients, there were no clinically significant changes in the size of the NIR lesions.

All AMN eyes (20 eyes from 11 patients) that were tested using the Humphrey (10 − 2, 24 − 2) visual field test revealed one or more paracentral scotomas. The shape and location of clinical fundus lesion closely matched the anomalies in the visual field. Wedge-shaped or circular visual field abnormalities were the most typical. (Fig. 1H1 and H2) At the final follow-up, 16 eyes showed persistent visual field scotomas.

Fluorescein angiography (FA) and indocyanine green angiography (ICGA) were performed on 7 patients. No abnormal fluorescence was observed in the FA and ICGA images of the AMN patients, which appeared to be normal (Fig. 1).

All patients underwent fundus autofluorescence (FAF) examination, but no abnormal fluorescence was detected in the areas of the lesions. The changes in FAF of all AMN patients were not as pronounced as the abnormalities seen in infrared or SD-OCT imaging (Fig. 2C1 and C2).

**Fig. 3:**
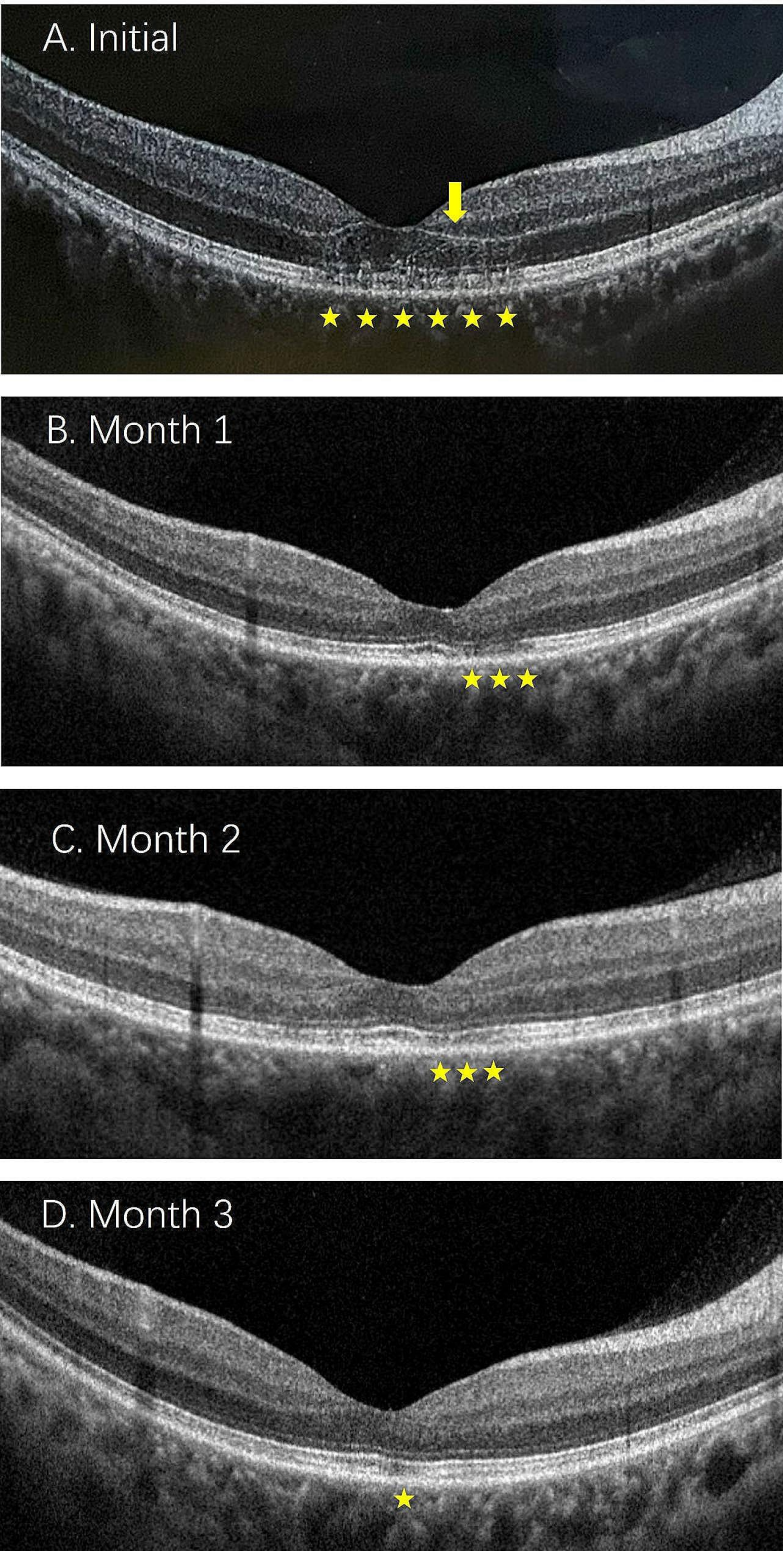
The follow up result of serial spectral-domain optical coherence tomography (SD-OCT) scans in the patient 2. Initial SD-OCT showed a hyperreflective band involving the outer plexiform layer (OPL) and outer nuclear layer (ONL) (yellow arrow) with disruption of the ellipsoid and interdigitation zones (yellow pentagram) (**A**). Serial SD-OCT scans obtained over the following 3 months showed partial improvement of the ellipsoid and interdigitation zones (yellow pentagram) (**B**-**D**)

All patients underwent SD-OCT. The SD-OCT findings in AMN patients showed one or more abnormal results, with the most common changes being the disruption of EZ (18 eyes), followed by OPL hyperreflectivity (4 eyes), ONL thinning (2 eyes), and Henle’s layer hyperreflectivity (1 eye). The time interval between the initial and final SD-OCT scans ranged from 8 to 20 weeks (average of 12.5 weeks). At the last visit, persistent changes observed on SD-OCT included ONL thinning in 10 eyes, partial reversal of EZ defects in 14 eyes, and persistent EZ defects in 6 eyes (Fig. 2 and 3).

**Fig. 4:**
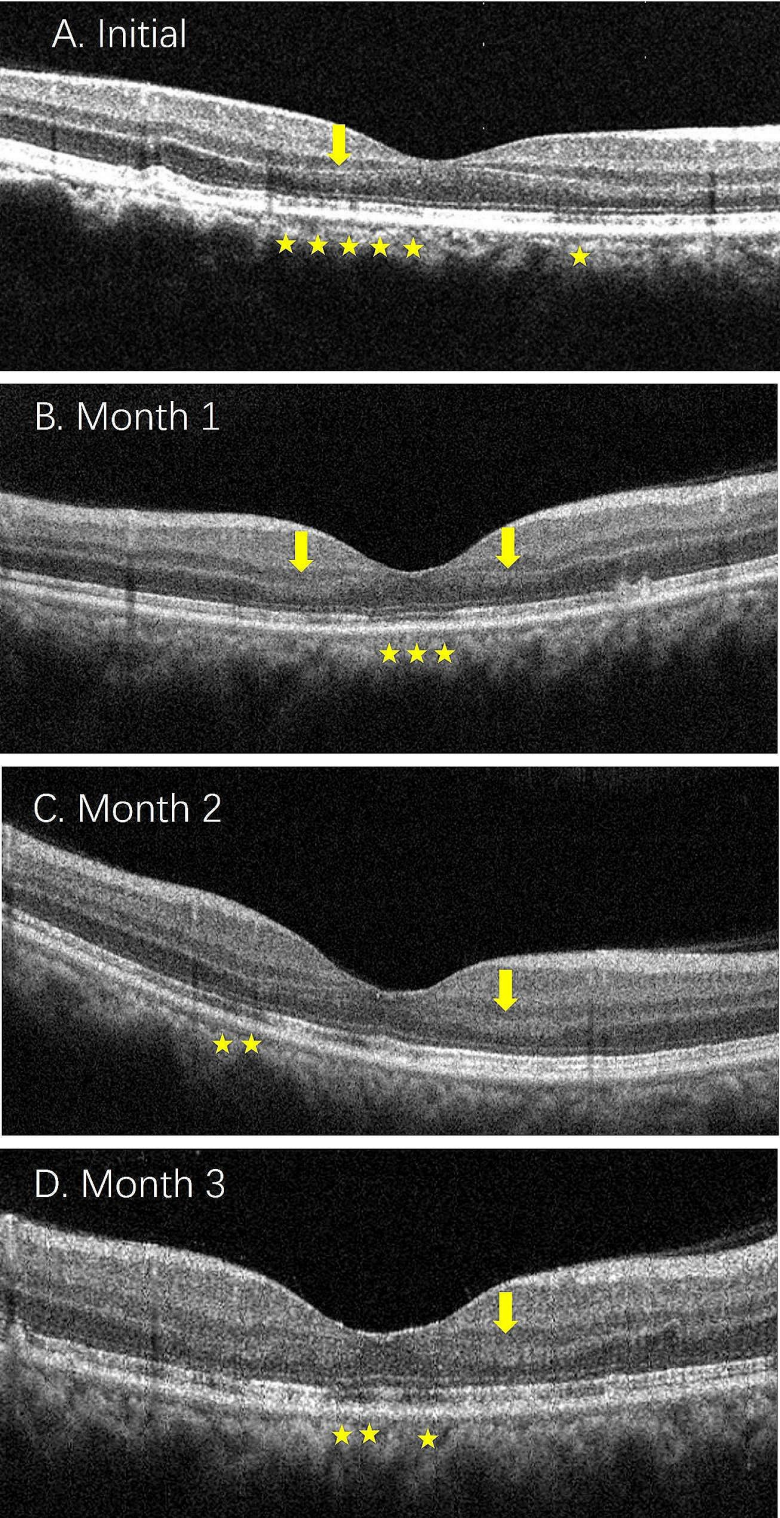
The follow up result of serial spectral-domain optical coherence tomography (SD-OCT) scans in the patient 7. Initial SD-OCT showed a hyperreflective band involving the outer plexiform layer (OPL) and outer nuclear layer (ONL) (yellow arrow) with disruption of the ellipsoid and interdigitation zones (yellow pentagram) (**A**). There was not significant improvement in the disruption of the ellipsoid and interdigitation zones (yellow pentagram) with persistence of hyperreflective areas within the OPL and ONL (yellow arrow) during the following 3 months (**B**-**D**)

OCTA examination was performed on 8 patients, showing mild attenuation of signal in the deep capillary plexus (DCP), corresponding to the hyporeflective areas observed in the NIR images. The superficial and middle capillary plexuses were normal (Fig. 4).

## Discussion

In COVID-19-induced extrapulmonary manifestations, ophthalmologists have observed a wide range of ocular manifestations associated with COVID-19, ranging from involvement of the anterior segment such as conjunctivitis and anterior uveitis to retinal and optic nerve diseases such as retinitis and optic neuritis [9, 10]. Some cases have reported COVID-19-related retinal lesions, mainly ischemic events such as flame-shaped retinal hemorrhages, cotton-wool spots, and wedge-shaped pale areas [11]. AMN is a rare retinal disease that has been reported to occur following dengue fever, influenza virus infection, and influenza vaccination, but the exact mechanisms underlying this association remain unknown. In our reported series of 11 AMN patients, they all developed AMN within days after COVID-19 infection, indicating a direct link between the onset of AMN and the pathogenesis of COVID-19 infection.

Consistent with previous research findings, the majority of AMN patients who developed AMN after COVID-19 infection in this case series were young females (average age of 33.8 years) [7]. The typical subjective complaints were scotomas, often accompanied by visual acuity decline and visual blurring. During our follow-up, most patients eventually recovered their visual acuity, but central and paracentral scotomas persisted in some patients. Similar to previous reports of AMN cases, visual acuity recovery typically took one to two weeks, while scotomas could persist for months. In the case report by Virgo and Ferkova, the patient’s primary symptoms were paracentral scotomas [12, 13]. Aidar [14] reported a 71-year-old woman with AMN in her left eye, occurring 14 days after the onset of COVID-19 symptoms. The patient presented with visual field scotomas and visual acuity was quite low at 0.5 LogMAR, and there was no improvement even after a 2-month follow-up.

This study retrospectively reviewed the results of various imaging techniques in AMN patients following COVID-19 infection, highlighting characteristic findings of AMN. In our case series, AMN lesions appeared as hyporeflective areas with distinct borders on NIR, located around the fovea of macula. Correlations between the photoreceptor layer disruption on SD-OCT and the hyporeflective anomalies found on NIR imaging may point to subclinical melanin alterations in the retinal pigment epithelium (RPE). When it came to identifying structural anomalies linked to AMN, SD-OCT was remarkably sensitive. The majority of reported eyes exhibited one or more abnormal findings, with ONL hyperreflectivity and EZ disruption being the most frequent. Even in situations where clinical examination and color fundus photography are unable to detect abnormalities, NIR imaging paired with SD-OCT may be the most effective imaging technique for identifying AMN.

SD-OCT scanning is also valuable in tracking structural changes. This case series found ongoing changes on SD-OCT, including thinning of the ONL and partial reversal of photoreceptor layer defects in some patients, but photoreceptor layer defects persisted without improvement in other patients. James et al. [15] described a case of a 22-year-old female referred for examination because she had bilateral scotomas and a mildly symptomatic COVID-19 infection. Over a 6-month follow-up, minimal improvement was observed in hyporeflectivity on near-infrared imaging and EZ defects on SD-OCT [15]. However, there have also been case reports of an older male (70 years old) developing AMN following COVID-19 infection, and at a 1-month follow-up, SD-OCT scanning of the macula showed thinning of the ONL with near complete restoration of EZ integrity [16]. In this case series, the SD-OCT abnormalities in the patients ONL partially resolved during the final follow-up, and EZ integrity did not fully recover to normal. The reasons for the varying outcomes in terms of retinal damage repair are currently unclear.

In this series of AMN cases, FA, ICGA and FAF imaging did not show characteristic changes. Although white dot syndromes like acute posterior multifocal placoid pigment epitheliopathy (APMPPE) and multiple evanescent white dot syndrome (MEWDS), which frequently affect young patients and have distinctive vascular imaging findings, may be another cause of acute vision loss, these imaging techniques may help exclude them out. Due to similarities in the affected population, AMN has traditionally been confused with white dot syndromes [17]. However, non-inflammatory ischemia of the deep retinal capillary plexus is the main cause of AMN.

Clinical evidence led to the first classification of AMN as a retinal illness; nevertheless, its pathogenesis is likely complex. The development of AMN has been linked to ischemia, inflammation, and infection, among other potential reasons. Turbeville et al. [18] suggested that vascular pathology may unify these different associations. Even though a vascular explanation for AMN has been suggested, there hasn’t been much proof up until lately. Since OCT angiography has been applied to investigate the pathophysiology of AMN, there has been evidence of involvement of the choroidal vasculature or the deep retinal capillary plexus. Previous studies have found reduced perfusion in the deep macular capillary plexus of AMN patients [5, 19], and empty flow voids have been observed in choroidal vasculature corresponding to hyporeflective areas seen on near-infrared imaging [20, 21]. In previous case reports regarding AMN following COVID-19 infection, it was observed that multiple regions exhibited reduced blood flow signals at the level of the deep capillary plexus corresponding to the region of the OCT and NIR abnormalities [22, 23]. However, OCTA changes are not always detected. In Dinh RH’s case series, only half of the patients on whom OCTA was performed had positive flow voids in the deep capillary plexus or choriocapillaris [24].

In our patients, evidence of empty flow voids in the deep retinal capillary plexus, which corresponded to hyporeflective areas on near-infrared imaging, was observed when patients were examined after experiencing visual symptoms. The primary receptor for SARS-CoV-2 entrance into host cells is thought to be angiotensin-converting enzyme 2 (ACE2) [25]. Studies have shown that ACE2 protein can be detected in the retina, suggesting its potential as a target for viral attack on retinal cells. Animal studies have also demonstrated penetration of SARS-CoV-2 through retinal cells [26]. However, the presence of SARS-CoV-2 infection in the retina of COVID-19 patients is still controversial and requires further histopathological analysis. Additionally, ACE2 inactivation mediated by SARS-CoV-2 may lead to an imbalance in the ACE Ang II/ACE Ang (1–7) axis, resulting in vasoconstriction. Studies have found endothelial dysfunction and a prothrombotic state in COVID-19, as well as the presence of coagulation abnormalities and antiphospholipid antibodies [27, 28]. This suggests that COVID-19-related vascular and inflammatory complications causing inadequate perfusion of the deep retinal capillary plexus may play a significant role in the pathogenesis of COVID-19-related AMN.

The main limitation of our study is the inability to confirm that AMN is secondary to COVID-19 infection. Although the patients included in our study developed AMN shortly after COVID-19 infection, we cannot definitively attribute AMN to COVID-19. While analysis of inflammatory biomarkers in aqueous humor and vitreous samples using RT-PCR could reveal immune mechanisms, invasive procedures were performed when necessary. Due to ethical considerations, none of our patients required intraocular sampling. Furthermore, our study is a retrospective observational study with a small sample size. In addition to above, the duration of follow-up in our study was relatively short, which averaged 12 weeks. A longer follow-up study is required to provide a more comprehensive picture of the disease’s course and its potential relationship with systemic factors, ultimately contributing to a more thorough understanding of AMN.

In conclusion, this case series found that COVID-19-related AMN predominantly affects young women. AMN is a rare but severe retinal disease that can result in persistent scotomas over a long-term course. With SD-OCT being sensitive in detecting retinal structural abnormalities and OCTA being essential in recognizing reduced blood flow in the deep capillary plexus, multimodal diagnostic imaging has offered increasingly comprehensive characteristics of AMN. For all patients experiencing visual difficulties during COVID-19 infection, we advise complete retinal evaluation, including attentive analysis of the macular OCT, in order to quickly detect the presence of AMN. Currently, research on COVID-19-related retinal diseases is still limited, and further studies are needed to understand the retinal pathophysiological changes caused by SARS-CoV-2.

## Data Availability

The datasets used and/or analyzed during the current study are available from the corresponding author on reasonable request.

## Abbreviations

AMN: acute macular neuroretinopathy
COVID-19: 2019 novel coronavirus disease
SARS-CoV-2: severe acute respiratory syndrome coronavirus 2
SUN: Standardization of Uveitis Nomenclature
RT-PCR: real-time reverse transcription polymerase chain reaction
BCVA: best corrected visual acuity
IOP: intraocular pressure
HM: hand movement
CF: counting finger
SD-OCT: spectral domain optical coherence tomography
OCTA: optical coherence tomography angiography
NIR: near-infrared reflectance
FFA: fundus fluorescein angiography
ICGA: indocyanine green angiography
FAF: fundus autofluorescence
OPL: outer plexiform layer
ONL: outer nuclear layer
EZ: disruption of the ellipsoid zone
